# Food-related taboos and misconceptions during pregnancy among rural communities of Illu Aba Bor zone, Southwest Ethiopia. A community based qualitative cross-sectional study

**DOI:** 10.1186/s12884-021-03778-6

**Published:** 2021-04-17

**Authors:** Dereje Tsegaye, Dessalegn Tamiru, Tefera Belachew

**Affiliations:** 1 Department of Nutrition and Dietetics, Institute of Health, Jimma University, Ethiopia, Jimma, Ethiopia; 2 College of Health Science, Department of Public Health, Mettu University, Metu, Ethiopia

**Keywords:** Food taboo, Misconception, Pregnancy, Ethiopia

## Abstract

**Background:**

Poor maternal nutrition adversely affects pregnancy and birth outcomes. In many societies, there are dietary restrictions due to misconceptions or food taboos during pregnancy which consequently results in the depletion of important nutrients. These cultural malpractices and beliefs can influence the dietary intake of pregnant women which subsequently affects the birth outcome. The study aimed at exploring the extent of food taboos and misconceptions during pregnancy in rural communities of Illu Aba Bor Zone, Southwest Ethiopia.

**Methods:**

A qualitative study was conducted using an in-depth interviews of key informants and focus group discussions among purposively selected pregnant women and their husbands, health care workers, health extension workers, and elderly people. Data were transcribed verbatim, thematized; color-coded, and analyzed manually using the thematic framework method.

**Result:**

Thorough reading and review of the transcripts generated three major themes. The primary theme was the belief and practice of taboos related to the intake of certain food items during pregnancy. Pregnant women, their husbands, and mothers-in-law believed that certain foods should be avoided during pregnancy. The second theme was foods that were held as taboo and the reason attached to it. The most common food items held as taboo were related to the consumption of vegetables like cabbage, pumpkin, milk and milk products, sugar cane, fruits like bananas and avocado and egg. The main reasons to avoid these foods were beliefs that it can be plastered on the fetal head, making fatty baby which is difficult for delivery. The third theme was the reasons underlying adherence to food taboos which is deeply embedded in the person’s believes and attitudes of the pregnant women, who were nested within the influence of the social environment surrounding them and the traditional beliefs and values of the society in general.

**Conclusions:**

The results showed a widespread practice of food taboos during pregnancy in the study area. The finding suggested that there is a need for strengthening the nutrition counseling components of antenatal care follow-up and planning comprehensive nutrition education through involving important others to dispel such traditional beliefs and prevent food taboo practices in the study community.

**Supplementary Information:**

The online version contains supplementary material available at 10.1186/s12884-021-03778-6.

## Background

Pregnancy is a period when physiological nutrient demands are considerably increased. This period imposes the need for considerable extra calorie and nutrient requirements. Therefore, a balanced and adequate diet is of paramount importance during pregnancy and lactation to meet the increased needs of the mother and to prevent “nutritional stress” [1]. Nevertheless, diet restrictions due to the mistaken belief or food taboos during the critical period of pregnancy may compromise the woman’s ability to meet the increased demands of the essential nutrients, hence putting the woman at an increased risk of adverse pregnancy outcomes [2].

The practice of food taboo is widespread in developing countries, while there is a variation in the type of food considered as taboo, and also the reasons attached to the taboos vary from society to society. For instance, in a study in South Africa, the most common foods that were avoided were meat and fish, potatoes, fruits, beans, eggs, butternut, and pumpkin, which are a good sources of in essential nutrients. The reasons for avoidance of the food items were associated with pregnancy outcome, labor and to avoid an undesirable body appearance for the baby [3]. Studies in Ethiopia also revealed that food items avoided were, linseed, honey, sugarcane, milk, Yoghurt, cheese, fatty meat, eggs, fruits, and vegetables, and reasons mentioned for the avoidance of these food items were it get plastered on the fetal head, makes fatty baby and difficult delivery, fear of abortion, evil eye, fetal abnormalities [4, 5]. Even though adequate dietary intake during pregnancy could be affected by many factors including affordability and accessibility, food taboo has been recognized as one of the factors contributing to maternal under-nutrition in pregnancy; especially in rural settings [6, 7].

Food taboos are closely associated with dietary intakes of pregnant women underscoring the need for assessing food taboos and related misconceptions during pregnancy to design appropriate interventions at national, regional, and local levels. However, studies in Ethiopia particularly that involve several actors that can influence food consumption like health workers, the elderly women, and health extension workers, as well as husbands of pregnant women are rare, if not nonexistent. Hence, this study used focus group discussions (FGDs) with pregnant women and their husbands, as well as key informant in-depth interviews (KIIs) with various actors to explore maternal dietary habits, food taboos, and misconceptions which influences the dietary intake of pregnant women in rural Ethiopia.

## Methods

### Study setting

A community based qualitative cross-sectional study was conducted in eight rural Kebeles (the lowest administrative unit) selected from four districts of Illu Aba Bor Zone, Southwest Ethiopia. The zone is one of the twenty-one zones in Oromia regional state. The Zonal town, Mettu is found at a distance of 600kms from the capital of Ethiopia to the southwest direction. There are 14 districts in the zone with a total population of 933,345 where 467,553 are males. The dominant means of livelihood in the Zone is agriculture and cereals such as maize, sorghum, millet, and legumes like beans and peas are the commonly grown crops. Fruits and vegetables also grow in the area and coffee is the main cash crop of the zone.

### Study design and participants

An exploratory study was conducted from May to June 2019 using a qualitative method mainly Focus group discussions (FGDs) and in-depth interviews of Key informants (KIIs). These qualitative methods were used to best explore the food taboos and dietary habits since they are sensitive issues and it is necessary to uncover the why and the how behind such practices. To thoroughly investigate the cultural and community factors related to food taboos during the prenatal period, four health care providers, four Health Extension Workers (HEWs), and four elders (mothers-in-law), a total of 12 key informants were interviewed. Eight FGDs (four with husbands and four with pregnant women) having 8–10 purposively selected participants were conducted. The number of in-depth interviews and FGDs were determined based on the level of information saturation which was determined by transcribing the discussions of each day’s session. Purposive sampling was used to select the KII and FGD participants. The criteria for FGD eligibility required that women should be pregnant, able to understand and speak the local language and give consent, and husbands of a pregnant woman who is also able to understand and speak the local language and give consent. Homogeneity was maintained by sex and education. The criteria for KII participants’ selections were being health care workers, health extension workers, and the elderly. The conduct and reporting of this study are compiled using the guidelines outlined in the consolidated criteria for reporting qualitative research (COREQ) [8]; all details are provided in the Additional file 1.

### Data collection methods and tools

Piloted interview guide questions were used for both the FGDs and IDIs (Additional file 2). All the participants were approached face-to-face and none of them declined to participate to the study. The FGDs were held with pregnant women and their husbands, separately so that they can express their opinions without fear of being arbitrated by their respective partners. The KIIs were conducted with health care workers, health extension workers, and the elderly (grandmothers /mothers-in-law). Both the FGDs and in-depth interviews were held at the nearby health posts and health centers. During the KIIs, the participants were encouraged to take an active role in establishing the flow of the interview. All questions used in the FGDs and KIIs were open-ended and new questions arising from the responses from the respondents, as participants were able to build on each other’s ideas and comments. Redundancy of responses was considered to be saturated and was removed every evening after transcribing the day’s work and preliminary analysis. New questions were added, whenever an information gap was identified. The data collection team was composed of the principal investigator (PI) and a research assistant, who had tertiary level qualifications. The principal investigator is a Ph.D student. The research assistant was a BSc holder in public health and trained before the actual data collection period. Before the commencement of the study, the research assistant did not have a relationship with the participants. However, participants were notified of the reasons for conducting the research in the study area. The principal investigator (PI) and one recorder/note-taker facilitated each focus group discussion (FGD) and key informant in-depth interviews (KIIs) with the participants. The data were audio-recorded and to assure triangulation of the data with the record, the team took note including memos of participant’s behavior and contextual aspects. The focus group discussions took a minimum of an hour and half whereas the indepth interviews took a minimum of half an hour.

### Trustworthiness

The quality of qualitative research is assured by meeting standards of trustworthiness through addressing credibility and transferability [9]. To satisfy credibility, participants from different districts and different backgrounds were included. Additionally, Focus group discusions, in-depth interviews, and field notes were used in the analysis of the data. The study provided descriptions of the setting, sample size and sampling procedure, eligibility criteria, interview processes, and findings to strengthen the transferability to different contexts. Validity and reliability were ensured by triangulation of the data gathered from the interviews with the information obtained at the FGD and then after sharing the results with the attendants.

### Data analyses

The tape-recorded FGDs supported by handwritten field notes were transcribed verbatim and analyzed manually using the principle of systemic text condensation [10]. Transcripts were reviewed repeatedly to gain a thorough sense of the overall content in the texts, to identify central meaningful units in the material, condense the content through color-coding of the text, and finally create categories that contain the condensed meaning of the main themes in the material. The data were then organized into themes. Sections of the discussions were quoted verbatim, and a few were modified to enhanced readability. The results were presented using narratives using the verbatim of the study participants as illustrations to substantiate major assertions. Quotes were translated from the local language (Afan Oromo) to the English language. Phases of the thematic analysis of the qualitative data is indicated in Table 1 below.

**Table 1:** Phases of the thematic analysis of the qualitative data, Illu Aba Bor Zone Southwest, Ethiopia, 2019

Phase	Description
Transcription of data	The audiotape interviews supported by handwritten notes were transcribed verbatim.
Familiarization with the data	Familiarization with the whole interview was made by listening to the audio recording and/or reading the transcripts and contextual or reflective notes that were recorded.
Generating initial codes	The transcripts were read carefully line by line applying a paraphrase or label (a code) to describe what has been interpreted in the passage as important.
Searching for themes	DTs reviewed and grouped codes according to the similarity of the topic and started to form potential themes. Theme names that captured all codes included within the theme were developed.
Reviewing themes	The themes were checked with the coded data extracts and assessed in terms of how well they represented the entire data set.
Defining and naming themes	The names and definitions of each theme were refined and a short description of each theme was developed by the principal investigator.
Producing report	A full report of the analysis was written by DTs with the use of the most appropriate extracts and relating to the original research question. The report was read and reviewed by all authors.

## Results

### Background characteristics of the study participants

A total of seventy-nine (79) respondents participated in the eight FGDs and Twelve KIIs. The participants comprise twenty-six pregnant women, twenty-four men (husbands of pregnant women), four health professionals, four health extension workers four elderly (mothers-in-law). The participants represented a wide age range (20–63 years) and the educational status of the respondents ranged from no formal education to secondary school and above. (Table 2).

**Table 2:** Socio-demographic characteristics of the participants in rural Illu Aba Bor Zone, Southwest Ethiopia, 2019

Characteristics of respondents	Categories	Frequency(n)	Percent (%)
Age range	20–63 years	–	–
Sex	Male	35	44.3
	Female	44	55.7
Educational status	No formal education	41	51.9
	Primary	20	25.3
	Secondary & above	18	22.8
Occupational status	Employee	21	26.6
	Housewife/Farmer	51	64.6
	Daily laborer	7	8.8

From a thorough review and readings of the scripts, the following three themes were identified:

- Beliefs and practice of food taboos in the community.
- Food items held as taboos and reasons attached to it
- Reasons nderlying the adherence to the food taboos and misconceptions

### Beliefs and practice of food taboos in the community

The respondents were asked whether they were aware of any foods that are culturally prohibited during pregnancy. Differing opinions were noted regarding the practice of food taboos.

Some pregnant women, their husbands, and mothers-in-law believed that some foods should be avoided during pregnancy to protect and support maternal health. A KII participant mother-in-law said,

*“…When they have morning sickness they cannot eat oily foods. Restriction of oily foods is*

*practiced early in pregnancy to reduce the likelihood and severity of morning sickness...”*

Similarly, another mother-in-law, explained the following corroborating what was said earlier,

*“… Intake of oily foods may be limited throughout pregnancy and that, in general, pregnant women don’t eat as much oily food...”*

FGD participant pregnant mother also stated,

*"…pregnant women should be careful and avoid certain foods, particularly towards the last*

*trimester. Our community strongly believes that what a pregnant woman eats in the last months of her pregnancy goes directly to the womb to feed the baby. Thus, some foods can hurt the fetus…”*

In contrast, the health care workers and health extension workers believed that food taboos are becoming an old story.

One KII participant said,

*“…I do not think many people still believe that pregnant women need to avoid some foods… except few women that live in far-off areas, …”*

Similarly, another KII participant detailed,

*“… ihhhhh, though in the former times there was the restriction of foods like egg, milk and milk products, due to the belief that it makes the fetus big and gets attached to the fetal body,… nowadays there is no such practice in our community…"*

Another KII participant also stated,

*“…most of our community members are now questioning the reason behind these taboos and the need for adherence, so the practice is not widely observed in the district ...”*

Another KII participant stated the following substantiating the same opinion,

*“... Educated people are not strongly upholding the taboo and beliefs…”*

### Foods held as taboos and reasons attached to it

Pregnant women, their husbands, and mothers-in-law believed that certain foods should be avoided during pregnancy (Table 3).

**Table 3:** Summary of the taboo food and reasons mentioned by the participants, Illu Aba Bor Zone, Southwest Ethiopia, 2019

Taboo foods	Reasons behind the taboo	Discussants who mentioned the reason
Oily foods	To reduce the likelihood and severity of morning sickness	Few of the KII and FGD participants
Banana	Plastered onto the fetal head	Most of the FGD and KII participants
Avocado	Plastered onto the fetal head	Nearly half
Taro	Plastered onto the fetal head	Few participants
Milk and milk products	Makes the baby big / Plastered onto the fetal head and body	Most of the FGD and KII participants
Egg	Makes the baby big/Difficult delivery	Most of the FGD and KII participants
Sugarcane	Excessive weight gain to the mother/ Makes the baby big / cause difficult delivery	Most of the FGD and KII participants
Cabbage	Causes abdominal cramp to the fetus after birth	Most of the KII and FGD participants
Pumpkin	Plastered onto fetal head and body	Few participants

FGDs across participant groups pointed to restriction of high carbohydrate foods particularly.

sugarcane. The consumption of this food was perceived to be associated with having bigger babies, which is believed to lead to difficult labor and delivery.

FGD participant and husband of pregnant mentioned,

*“…Our community strongly believes that if a pregnant woman eats sugarcane, she may have a big baby which endangers her life by making labor difficult, but I doubt the effect …”*

Similarly, FGD participant pregnant women stated,

*“…if you eat these kinds of foods or meals you will have difficulty during birth. Consumption*

*of “a lot of sugar” during pregnancy leads to an increase in weight and a risky delivery*

*as the increase in weight during pregnancy makes the baby very big…”*

Similarly, the discussants’ reported that foods like fruits specifically banana and avocado, and some types of vegetables like cabbage, pumpkin, and taro (Colocacia esulenta) are considered as taboo for pregnant women particularly as the gestational age advances. The reason attached to the taboo of these foods as stated by the discussants is that cabbage may cause abdominal cramps to the baby when born, whereas pumpkin, banana, Avocado, and Taro (Colocacia esculenta) locally named “Godare” are believed to pass to the fetus in the womb and get plastered to the head of the fetus. In explaining this instance a mother in law, KII participant stated,

*“….if a pregnant woman consumes banana, avocado and taro (locally known as Godare) particularly as the gestational age advances; it can pass to the womb and get attached to the baby’s body while cabbage causes abdominal cramp to the fetus after birth. …”*

Likewise, FGD participant husband of a pregnant woman uttered,

*"… I heard some people saying that pregnant women should not consume pumpkin... But I*

*do not know the reason…”*

By the same token, the consumption of dairy products (milk, yogurt, cheese) and eggs during pregnancy is considered harmful to the fetus and the mother. One of the FGD participant pregnant women said,

*"….it is believed that pregnant women should avoid consuming dairy products like yogurt and cheese, particularly in the last weeks of her gestation. This is because dairy products can pass to the womb and attach to the baby’s body…”*

One of the respondents had the following to say, which reaffirms the assertions of the other discussants stated above:

*“…A pregnant woman shouldn't eat some foods such as eggs. This is because the fetus will become very big and the mother will have difficult labor and delivery...”*

### Reasons underlying the adherence to the food taboos

The underlying reasons for adherence to the food taboos from explanations provided by study participants were grouped into three broad categories: cultural influence, social context, and beliefs of the pregnant women themselves.

### Cultural influences

The majority of the study participants viewed the existence of traditional practices and beliefs about foods held as taboos are inherent in the community. They stated the reasons for their practice of food taboos stem from cultural influences. One FGD participant, husband of pregnant women, stated the following to elaborate the matter,

*“… Our community strongly believes that if a pregnant woman consumes foods that are held*

*as taboo, she may have a big baby which makes labor difficult and endanger her life …”*

### Social context

The pressure from important others surrounding the pregnant women is a critical driver of the adherence to the food taboos. The discussant expressed that husbands and mothers-in-law impose cultural and traditional beliefs on pregnant women. One FGD discussant also mentioned peer influence. To substantiate this state affair, a KII participant Mother-in-law, stated,

*"….pregnant women should avoid foods like banana, avocado, and taro (locally known as Godare) …if she consumes particularly as the gestational age advances; it can pass to the fetus in the womb and gets attached to the baby’s body …”*

Similarly, one pregnant woman indicated,

*“… It is believed that some foods can pass to the fetus in the womb and plastered on the fetal body…and women laugh at each other if a woman gives birth to a baby full of the white substance on the body…that is why we follow the food restrictions…"*

### Attitudes and beliefs of the individual pregnant women

This study revealed that recipients of the cultural practices, pregnant women, were without an understanding of why they do what they do. However, some of the discussants believed that consumption of the foods held as taboo may hurt the fetus. A pregnant woman mentioned,

*"… if you eat certain kinds of foods or meals you will have difficulty during birth. …”*

Similarly, another pregnant woman, FGD participant stated,

*“… it is believed that consumption of the tabooed food may cause damage to the fetus, and … I do not want my baby hurt, that is why I avoid the foods held as taboo…”*

**Fig. 1:**
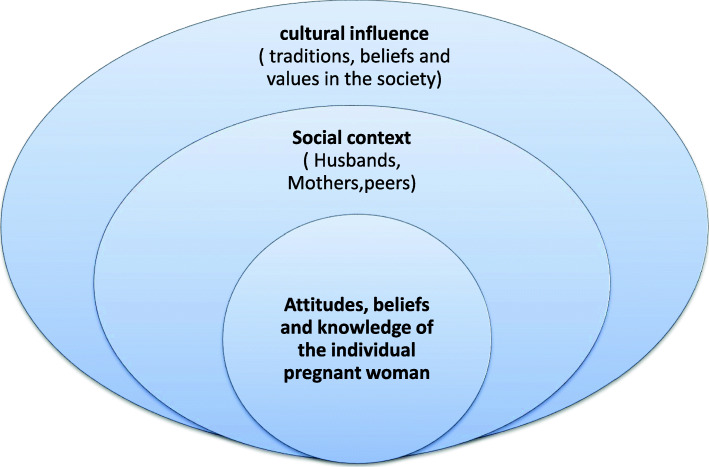
The interrelationships between drivers at the different levels underlying reasons for the adherence to the food taboos and misconceptions during pregnancy in rural communities of Illu Aba Bor Zone, South West Ethiopia

The reasons for the food taboos are deeply embedded in the person’s believes and attitudes of the pregnant women, who are nested within the influence of the social environment surrounding her (important others) and the traditional beliefs and values of the society in general. The interrelationships between drivers at the different levels are illustrated in Fig. 1.

## Discussion

The study found out that one or more foods are avoided during pregnancy as a result of food taboos. This finding is supported by many studies reported elsewhere, where women would adhere to different food taboos and beliefs [5, 11–14]. The foods items most frequently avoided during pregnancy were dairy products like milk, cheese, and yogurt, fruits like bananas, Avocado, eggs, vegetables like cabbage, pumpkin, Taro, and sugarcane. Most of the foods that were reported as taboo are rich sources of essential micronutrients, which are crucial for maternal health and child growth and development. Findings from the current study support others in which food taboos during pregnancy were found to be more elaborate, nutritionally significant, and differ only in the type and reasons attached to avoidance of the food type [4, 5, 15–17].

The main reasons for the restriction of the food items held as taboo in this study were, the fear that the food will be plastered on the fetal head and having a big baby which make labor and delivery difficult. This finding is supported by findings from a study in Shashmane and Addis Ababa that state the reasons for adhering to pregnancy-related food taboos and myths to be a large baby and difficult birthing and ‘food sticking’ on the fetal head [4, 12]. A study in Accra Ghana also showed that pregnant women were restricted from consuming certain foods to check their health, control the weight of the expectant mother and unborn child and ensure there is a safe delivery, which is consistent with our study finding [6].

The study revealed that sugarcane is commonly restricted during pregnancy because it is believed to causes excessive weight gain and difficult deliveries. A similar finding was reported from a study in Arsi Zone, where the discussants considered consumption of sugarcane to be associated with having bigger babies, which is believed to lead to a difficult delivery [5]. Similarly, a study in Ghana showed that consumption of sugary foods makes fetus large [17].

The study further revealed the community belies the fact that if pregnant women consume fruits like bananas or avocado and vegetables such as pumpkin and taro particularly towards the last weeks of her pregnancy, it passes to the fetus in the womb and plastered onto the head of the fetus. This finding is supported by the report from Ghana and East Gojam, Ethiopia in which consumption of bananas during pregnancy is believed to be attached to the head of the fetus [11, 17].

Another type of food considered taboo in the study area was the consumption of Eggs during pregnancy because it is believed that it makes the fetus large, contributing to a difficult delivery. This finding is again supported by the findings from the study in Ghana and Kenya that showed consumption of Eggs makes the fetus large [17, 18]. This finding is again supported by the study finding from Nigeria where consumption of eggs is prohibited during pregnancy because it is feared that the children may develop bad habits after birth [16]. The difference supports the literature that indicates there is no single reason but several reasons for the belief and adherence to food taboos [19].

Consumption of milk and milk products during pregnancy was also considered taboo in the study area. The reason for the restriction of the food type is that it passes to the fetus and gets plastered onto the head of the fetus and makes the baby big. Our study finding is supported by the study finding from Kenya which states the reason for the restriction of milk during pregnancy is due to the belief that it makes the baby bigger [18]. Similarly a study in Abala district of Afar region, Ethiopia also identified that consumption of milk and yogurt is considered taboo and the reason attached to the food held as taboo was to prevent the fetus from getting large and reduce the risk of long labor [15].

Another food type considered taboo during pregnancy was the consumption of leafy vegetables like cabbage which are considered to hurt the fetus and pregnant women. This finding is similar to the study report from Arsi Zone, Central Ethiopia that showed if a pregnant woman eats leafy vegetables, especially after 8 months of gestation, the leaf passes to the womb and attaches to the baby’s head and forms what they called “particles” [5].

The study further identified that the explanations underlying adherence to food taboos provided by study participants were cultural influence; traditions, beliefs, and values in the society. This finding is consistent with the finding from the study in Tamilnadu state which showed that women were duty-bound to avoid specific food items due to cultural and traditional views [14]. Similarly, another study in Accra Ghana supports this finding that the main reason for belief and adherence to food taboo was culture [6]. Another reason for the adherence to the food taboos was the social environment; influence from mothers-in-law and peers). This finding agrees with the finding from a study in West Bengel in which the majority of the respondents pointed that their mother, mother-in-law, other senior female members, and female neighbors were the advisors of those taboos [13]. Attitude and beliefs of the pregnant women themselves towards the food taboos is another reason for the adherence to the food taboos. Similarly, a qualitative study conducted in Addis Ababa points that the underlying reasons for the adherence to pregnancy-related food taboos and myths (PRFT) were largely traditionally held beliefs and misconceptions [12]. The findings have strong practical implications. After several years of implementing the national nutrition program (2008), national maternal and infant and young child feeding guidelines (2005), and deployment of both urban and rural health extension workers, the finding of pregnant women who practice food taboos indicates the necessity for revamping the implementation to the grassroots level. Especially, the fact that this practice is backed by the pressure from the social environment (important others) and is deeply embedded in the traditional believes of the society heralds the need for galvanizing more public health interventions to optimize the dietary practices of pregnant women in the study area.

The following limitations need to be considered when interpreting the findings of this study. Although the KIIs and FGDs were conducted with great care, the extent of over-reporting or underreporting of positive or negative behaviors may not be known. Moreover, purposive sampling was used to select the key informants and focus group discussant. Thus, the findings should not be generalized to the entire study population and beyond, but should be taken as an indication that taboos and misconceptions are still present among a number of the studied subjects.

## Conclusions

This exploratory study revealed that pregnant mothers in the study area are influenced by food taboos based on cultural perceptions and beliefs, with the fear of increasing body weight of the fetus which can result in either the mother facing problems during childbirth or the child will be born with ill health.

The most common foods prohibited as taboo were milk and milk products and eggs, some vegetables like cabbage and pumpkin, taro, banana from fruits, and sugarcane. Omitting those food staff from the requirement during pregnancy will have a long-term impact on the mother and fetus making maternal and child that is, for the mother and underweight during delivery of the infant and easily susceptible to disease during childhood.

Based on the finding of the study, we recommend nutritional counseling with emphasis during ant-natal care, and post natal service is imperative. At the same time, a comprehensive nutrition education involving significant others is recommended.

## Supplementary Information

**Additional file 1.**

**Additional file 2.**

## Data Availability

The datasets generated and analyzed during the current study are not publicly available because they are confidential to protect the participants’ anonymity but are available from the corresponding author on reasonable request.

## Abbreviations

FGD: Focus Group Discussion
HEW: Health Extension Worker
KII: Key Informant in-depth Interview
MCH: Mother and Child Health
PRFT: Pregnancy-Related Food Taboos and Myths
