# Supplementary material for: Food-related taboos and misconceptions during pregnancy among rural communities of Illu Aba Bor zone, Southwest Ethiopia. A community based qualitative cross-sectional study
Source: BMC Pregnancy Childbirth. 2021 Apr 17;21:309. doi: 10.1186/s12884-021-03778-6 (PMC8052673; doi:10.1186/s12884-021-03778-6)
Supplement: Supplementary file 1 — Additional file 1. [file 12884_2021_3778_MOESM1_ESM.docx]

| **COREQ (Consolidated criteria for Reporting Qualitative research) Checklist** | | | | |
| --- | --- | --- | --- | --- |
| **Topic** | Item **No** | | **Guide Questions/Description** | **Reported on Page No.** |
| **Domain 1: Research team and reflexivity** | | | |  |
| ***Personal characteristics*** |  |  | |  |
| Interviewer/facilitator | 1 | Which author/s conducted the interview or focus group? | | 5 |
| Credentials | 2 | What were the researcher’s credentials? E.g. PhD, MD | | 1 &5 |
| Occupation | 3 | What was their occupation at the time of the study? | | 1 &5 |
| Gender | 4 | Was the researcher male or female? | | Male |
| Experience and training | 5 | What experience or training did the researcher have? | | 1&5 |
| ***Relationship with participants*** | | | |  |
| Relationship established | 6 | Was a relationship established prior to study commencement? | | 5 |
| Participant knowledge of the interviewer | 7 | What did the participants know about the researcher? e.g. personal goals, reasons for doing the research | | 5 |
| Interviewer characteristics | 8 | What characteristics were reported about the inter viewer/facilitator? e.g. Bias, assumptions, reasons and interests in the research topic | | 5 |
| **Domain 2: Study design** | | | |  |
| *Theoretical framework* |  |  | |  |
| Methodological orientation and Theory | 9 | What methodological orientation was stated to underpin the study? e.g. grounded theory, discourse analysis, ethnography, phenomenology, content analysis | | 6 |
| ***Participant selection*** |  |  | |  |
| Sampling | 10 | How were participants selected? e.g. purposive, convenience, consecutive, snowball | | 4 |
| Method of approach | 11 | How were participants approached? e.g. face-to-face, telephone, mail, email | | 5 |
| Sample size | 12 | How many participants were in the study? | | 7 |
| Non-participation | 13 | How many people refused to participate or dropped out? Reasons? | | None |
| Setting of data collection | 14 | Where was the data collected? e.g. home, clinic, workplace | | 5 |
| Presence of non-participants | 15 | Was anyone else present besides the participants and researchers? | | No |
| Description of sample | 16 | What are the important characteristics of the sample? e.g. demographic data, date | | 7 |
| ***Data collection*** |  |  | |  |
| Interview guide | 17 | Were questions, prompts, guides provided by the authors? Was it pilot tested? | | 5 |
| Repeat interviews | 18 | Was repeat interviews carried out? If yes, how many? | | No |
| Audio/visual recording | 19 | Did the research use audio or visual recording to collect the data? | | 5 |
| Field notes | 20 | Were field notes made during and/or after the interview or focus group? | | 5 |
| Duration | 21 | What was the duration of the inter views or focus group? | | 5 |
| Data saturation | 22 | Was data saturation discussed? | | 5 |
| Transcripts returned | 23 | Were transcripts returned to participants for comment and/ or correction? | | Yes |
| **Domain 3: analysis and findings** | | | |  |
| ***Data analysis*** |  |  | |  |
| Number of data coders | 24 | How many data coders coded the data? | | 6 |
| Description of the coding tree | 25 | Did authors provide a description of the coding tree? | | (Table 2) page 6 |
| Derivation of themes | 26 | Were themes identified in advance or derived from the data? | | 6 |
| Software | 27 | What software, if applicable, was used to manage the data? | | No |
| Participant checking | 28 | Did participants provide feedback on the findings? | | 6 |
| ***Reporting*** |  |  | |  |
| Quotations presented | 29 | Were participant quotations presented to illustrate the themes/findings? Was each quotation identified? e.g. participant number | | Yes, 8 - 13 |
| Data and findings consistent | 30 | Was there consistency between the data presented and the findings? | | Yes |
| Clarity of major themes | 31 | Were major themes clearly presented in the findings? | | Yes, 8 - 13 |
| Clarity of minor themes | 32 | Is there a description of diverse cases or discussion of minor themes? | | No |

**Developed from:** Tong A, Sainsbury P, Craig J. Consolidated criteria for reporting qualitative research (COREQ): a 32-item checklist for interviews and focus groups. Int J Qual Health Care. 2007; 19:349–57
