# Supplementary material for: Food-related taboos and misconceptions during pregnancy among rural communities of Illu Aba Bor zone, Southwest Ethiopia. A community based qualitative cross-sectional study
Source: BMC Pregnancy Childbirth. 2021 Apr 17;21:309. doi: 10.1186/s12884-021-03778-6 (PMC8052673; doi:10.1186/s12884-021-03778-6)
Supplement: Supplementary file 2 — Additional file 2. [file 12884_2021_3778_MOESM2_ESM.docx]

**FGD and KII study guide**

1. What are the staple foods in this area?
2. What are the main factors which determine the choice of foods eaten?
3. Are there any special foods for pregnant women? If yes, which ones are they?
4. When are the above foods consumed? During pregnancy/immediately after delivery?
5. Are there any food taboos in this community?

Probe: During pregnancy?

1. Why should women observe food taboo during pregnancy period?
2. What type of food? (List them) Probe further:
3. What do you know will happen if they eat them? Probe: Reasons attached to consumption of the food held as taboo (Record for each food given)
4. Do they eat these foods outside pregnancy?

Probe: Before/After birth?

1. What are some of the experiences that you are aware of that women encounter when they don’t observe food taboo during pregnancy?
2. How did you get to know about food taboo during pregnancy? Probe:
3. How long have you known about Food Taboo?
4. Who first told you about Food Taboo?
5. What is the current level of Food Taboo adherence in this area? Probe: Explain.
6. To the best of your knowledge, are all pregnant women observing Food Taboo in this district?
7. Do you think that pregnant women should adhere to food taboos?

Probe: Husband/Mother/mother in law

1. What do you think will happen to them if they refuse to observe the Food Taboos?

Probe: Father (Husband)

1. What are the underlying reasons for adhering to food taboos during pregnancy?
2. Other than Food Taboo, are there other taboos and beliefs that women must hold during pregnancy?

Probe further if any: What are they?
